# Association of Relapse with Renal Outcomes under the Current Therapy Regimen for IgA Nephropathy: A Multi-Center Study

**DOI:** 10.1371/journal.pone.0137870

**Published:** 2015-09-15

**Authors:** Yanhong Yuan, Xiajing Che, Zhaohui Ni, Yifei Zhong, Yinghui Qi, Xinghua Shao, Qin Wang, Liou Cao, Minfang Zhang, Yuanyuan Xie, Chaojun Qi, Lei Tian, Shan Mou

**Affiliations:** 1 Department of Nephrology, Molecular Cell Lab for Kidney Disease, Ren Ji Hospital, School of Medicine, Shanghai Jiao Tong University, 160 Pujian Road, Shanghai 200127, China; 2 Department of Nephrology, Longhua Hospital, Shanghai University of Traditional Chinese Medicine, 725 South Wanping Road, Shanghai 200032, China; 3 Department of Nephrology, Punan Hospital of Shanghai, Pudong New District, Shanghai, 200125, China; Radboud University Medical Center, NETHERLANDS

## Abstract

**Background and Objectives:**

Renal relapse is a very common manifestation of IgA nephropathy (IgAN). The clinical characteristics and long-term outcomes of this condition have not yet been carefully explored.

**Design and Patients:**

Patients with biopsy-proven IgAN between January 2005 and December 2010 from three medical centers in China was a primary cohort of patients. From January 2010 to April 2012, data of an independent cohort of IgAN patients from Ren Ji Hospital, Shanghai, China was collected using the same inclusion and exclusion criteria. These patients formed the validation cohort of this study.

**Results:**

Of the patients with biopsy-proven IgAN from three medical centers, 489 patients achieved remission within 6 months following the therapy. Additionally, 76 (15.5%) of these patients experienced a relapse after achieving remission. During the median follow-up period of 66 months, 6 patients (1.4%) in the non-relapse group experienced renal deterioration, compared with 22 patients (29.6%) in the relapse group. Our study indicated that each 1-mmHg increase in the baseline diastolic blood pressure (DBP) was associated with a 4.5% increase in the risk of renal relapse; additionally, the male patients had a 3.324-fold greater risk of relapse compared with the female patients according to the adjusted multivariate Cox analysis. The nomogram was based on 489 patients achieved remission. The predictive accuracy and discriminative ability of the nomogram were determined by concordance index (C-index) and calibration curve. The results were validated using bootstrap resampling on the validation cohort.

**Conclusions:**

This study demonstrated that renal relapse is a potential predictor of prognostic outcomes in patients under the current therapeutic regimens for IgAN. And male patients with higher diastolic blood pressure had a greater risk of experiencing relapse.

## Introduction

Primary IgA nephropathy (IgAN) is the most common form of worldwide and is the main cause of end-stage renal disease (ESRD) in patients with primary glomerular disease [1, 2]. However, the pathogenesis of this disease remains unclear; thus, there are many patients who have frequent relapses or are treatment-resistant with decreasing renal function [3]. IgAN, therefore, remains a disease with a poor prognosis [1, 4, 5].

Many studies [6–8] have assessed the impact of proteinuria reduction in IgAN and the clinical relevance of defining partial or complete remission in this disease as a valuable prognostic indicator for both the clinician and the patient; therefore, the persisting remission state is an acceptable surrogate endpoint with which to assess the overall efficacy of treatment in IgAN patients.

The prevalence and significance of disease relapse and the impact of available treatments are poorly understood. Many studies have been published on predictive factors related to renal progression in IgAN [3, 9–11], but there are few data on predictive factors related to relapse. Most published studies are small, single-center investigations, and outcome data are often difficult to generalize to populations that are demographically diverse [9]. Therefore, regular monitoring for renal progression in patients who experience relapse is warranted. Identifying factors that induce relapse can be clinically useful in the management of IgAN patients.

The aims of this study were to identify risk factors for the occurrence of a renal relapse in adult patients with IgAN, and ascertain if renal relapse is a prognostic factor for long-term outcome in IgAN.

## Materials and Methods

### Patients

A retrospective study was conducted on a primary cohort of 903 patients with biopsy-proven IgAN who were recruited between January 2005 and December 2010 from three medical centers in China. All the patients had definite pathological data with a predominant mesangial deposition of IgA with a grade of at least 1+ detected by immunofluorescence staining and electron-dense deposits within the mesangium detected by electron microscopy. The exclusion criteria were as follows: age < 18 years (n = 30); follow-up duration < 36 months (n = 160); pregnancy (n = 3); systemic inflammation, such as Henoch-Schönlein purpura (n = 6); chronic advanced liver disease (n = 35); or atypical forms of IgAN (n = 12) [acute kidney injury (AKI) associated with macroscopic hematuria and crescentic IgAN]. A total of 657 patients were included.

From January 2010 to April 2012, data of an independent cohort of IgAN patients from Ren Ji Hospital, Shanghai, China was collected using the same inclusion and exclusion criteria.These patients formed the validation cohort of this study.

The study was approved by the Ethics Committee of Ren Ji Hospital, and all the participants provided written informed consent. All the kidney biopsy slides were reviewed by an experienced renal pathologist.

### Data collection

The patients had regular follow-up visits at intervals of at least 3–6 months. All the data were collected prospectively. Using the database records, the demographic and clinical data were reviewed for age, gender, baseline systolic blood pressure (SBP) and diastolic blood pressure (DBP), medical history, medications, follow-up duration, time to remission, time to endpoint, and responsiveness to treatment. The laboratory data obtained at the time of diagnosis of IgAN and during the follow-up included 24-hour urinary protein excretion (UPE), hematuria (uRBC/HP), serum creatinine (SCr), serum uric acid (UA), blood urea nitrogen (BUN), albumin (ALB), and total cholesterol levels.

The estimated glomerular filtration rate (eGFR) was calculated using the Modification of Diet in Renal Disease (MDRD) study equation: eGFR (ml/min/1.73 m^2^) = 180 × [SCr (mg/dl)]^-1.154^ × (age)^-0.203^ × (0.742 if female) [12]. Chronic kidney disease (CKD) was classified based on the Kidney Disease Outcomes Quality Initiative (K/DOQI) practice guidelines. The mean arterial blood pressure (MAP) was defined as the DBP plus one-third of the SBP. The proteinuria reduction ratio (UPE ratio) was calculated by the following equation: (data at follow-up—baseline data) / baseline data. The average UPE was determined for each 3-month block during the follow-up, and the average of the UPE values for every 3-month period within the first six months before remission (the baseline, the third month and the sixth month) was represented by the time-averaged UPE (TA-UPE) [13, 14]. The TA-ALB, TA-SCr, and time-averaged eGFR (TA-eGFR) values were calculated using the same method as that used to calculate the TA-UPE. The maximum levels of UPE and SCr during the follow-up period were recorded as the peak UPE and peak SCr levels, respectively. Similarly, the minimum levels of ALB and eGFR during the follow-up period were recorded as the minimum ALB and minimum eGFR, respectively. The renal lesions were graded according to Lee’s classification at the time at which the database was established [15].

### Definitions

The treatment response was evaluated at the sixth month after the initiation of therapy [16]. Complete remission (CR) was defined as a UPE < 0.3 g/d, along with normalization of all biochemical findings and a lack of worsening of renal function at the sixth month. Partial remission (PR) was defined as at least a 50% reduction in UPE at the sixth month compared with baseline. No response (NR) was defined as a < 50% reduction in UPE or an increase in UPE with or without renal deterioration after receiving six months of therapy [3]. Relapse was defined as the reappearance of significant proteinuria, defined as > 1.0 g/d and as a UPE increase of 50% from the lowest level of proteinuria after the start remission[3, 17]. The primary endpoint was a doubling of the baseline serum creatinine concentration; the secondary outcomes were ESRD and death. The patients were classified with progression when their eGFR values decreased by more than 50% or when they reached the endpoint during the follow-up period; the patients who exhibited stable renal function, defined as an eGFR that remained within 50% of the initial value, were considered to be non-progression patients [9].

### Statistical analysis

Statistical analyses to identify risk factors were performed using SPSS software (version 13: SPSS, Chicago, IL). The normally distributed variables were expressed as the means ± SD and compared using a t-test or analysis of variance (ANOVA) as required. The non-parametric variables were expressed as the median and range and compared using either the Mann-Whitney U test or the Kruskal-Wallis test. The chi-square test was employed for the categorical variables. The renal survival times were calculated from the first clinical assessment suggestive of renal disease to the last follow-up point. The relationships between the parameters and renal survival were assessed using Cox regression. The multivariate models used a stepwise forward selection procedure based on a likelihood ratio test with P > 0.10 for the removal and P < 0.05 for the inclusion of the variables, based on which a prognostic index (PI) was made [18]. Cumulative survival curves were derived using the Kaplan-Meier method, and the differences between survival curves were compared using the log-rank test. A nomogram was formulated based on the results of multivariate analysis and by using the package of *rms* in R version 3.2.1 (https://www.r-project.org/). The performance of the nomogram was measured by concordance index (C-index) and assessed by comparing nomogram-predicted versus observed Kaplan-Meier estimates of survival probability. Bootstraps with 1,000 resample were used for these activities. During the external validation of the nomogram, the total points of each patient in the validation cohort were calculated according to the established nomogram, then Cox regression in this cohort was performed using the total points as a factor, and finally, the C-index and calibration curve were derived based on the regression analysis. All tests were two-tailed, with P-values < 0.05 considered statistically significant.

## Results

### Clinical Characteristics

**Fig 1:**
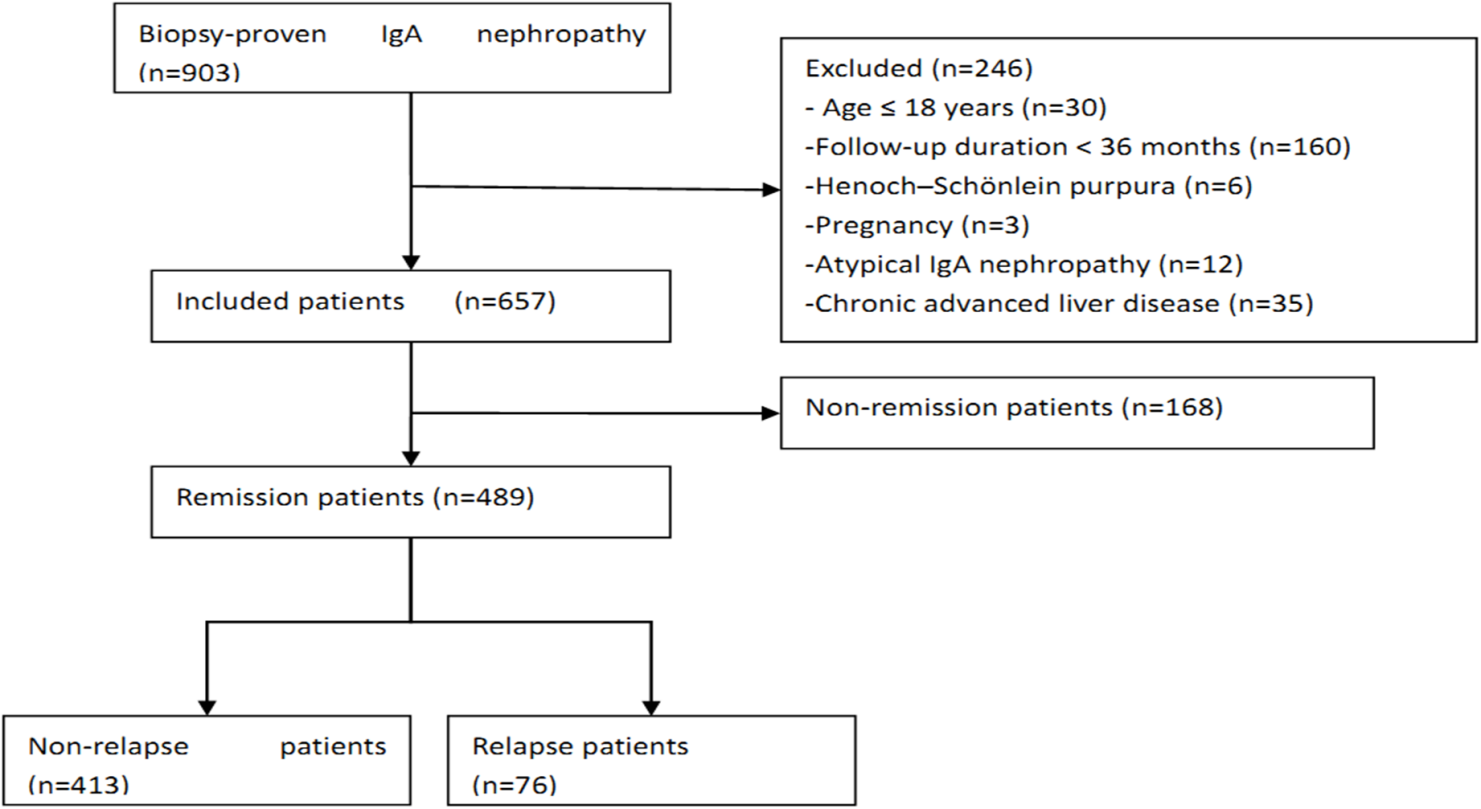
A flow diagram of the study.

From 2005 to 2010, 903 patients with primary IgAN were recorded in the registry database, 246 of whom were excluded and 657 of whom were included in this study (Fig 1). The median and interquartile range (IQR) of the follow-up time was 66 and 46–99 months, respectively. For the validation cohort, we studied 199 consecutive patients. The clinical characteristics of patients in the primary and validation cohorts at baseline and during follow-up are listed in Table 1.

**Table 1:** Demographics and Clinicopathologic Characteristics of Patients with IgAN.

Demographic or Characteristic	Primary Cohort n = 657	Validation Cohort n = 199
**Baseline**		
Age (years)	38.06 ± 12.27	38.14 ± 12.31
Gender: Female n (%)	360 (54.8)	112 (56.3)
SBP (mmHg)	125.49 ± 16.37	126.03 ± 17.88
DBP (mmHg)	80.67 ± 11.12	82.20 ± 12.39
MAP (mmHg)	95.61 ± 11.92	96.81 ± 13.23
SCr (μmol/L)	86.25 (67.50–117.10)	89.80 (69.00–115.10)
eGFR (ml/min/1.73 m2)	74.91 (54.01–100.37)	73.49 (54.42–98.37)
BUN (mmol/L)	5.85 (4.61–7.75)	6.30 (5.20–8.00)
UA (μmol/L)	364.50 (310.50–434.25)	374.00 (315.00–443.00)
Hb (g/L)	132.00 (122.00–145.25)	131.50 (121.00–145.75)
ALB (g/L)	38.80 (34.53–41.28)	39.30 (35.63–41.50)
UPE (g/d)	1.78 (1.06–2.96)	1.53 (1.00–3.02)
uRBC /HP	25.00 (7.00–65.00)	21.35 (5.70–46.15)
CKD stage n (%)		
Stage 1	232 (35.3)	65 (32.7)
Stage 2	219 (33.3)	71 (35.7)
Stage 3	183 (27.8)	57 (28.6)
Stage 4	20 (3.2)	4 (2.0)
Stage 5	3 (0.4)	2 (1.0)
Renal biopsy, Lee’s classification n (%)		
Grade I	12 (1.9)	0
Grade II	12 (1.9)	9 (4.5)
Grade III	316 (48.1)	94 (47.2)
Grade IV	266 (40.4)	77 (38.7)
Grade V	51 (7.7)	19 (9.5)
ACEI/ARB	104 (15.9)	32 (16.1)
Steroids	353 (53.7)	101 (50.8)
Immunosuppressors	200 (30.5)	66 (33.2)
**Follow-up**		
Remission n (%)	489 (74.4)	157 (78.7)
Complete remission n (%)	191(29.1)	52(26.1)
Partial remission n (%)	298 (45.3)	105 (52.8)
Non-response n (%)	168 (25.6)	42 (21.3)
Relapse n (%)	76 (15.5)	28 (17.8)
Length of follow-up (months)	66 (46–99)	31 (22–56)
UPE ratio within 3 months	0.53 (0.19–0.75)	0.47 (0.19–0.71)
UPE ratio within 6 months	0.65 (0.38–0.82)	0.57 (0.24–0.80)
ALB g/L at month 3	40.95 (37.18–44.53)	41.10 (37.93–44.98)
ALB g/L at month 6	42.40 (38.95–45.30)	42.40 (39.90–45.30)
TA-ALB (g/L)	42.23 (38.82–44.89)	42.58 (40.04–44.72)
Minimum ALB (g/L)	37.40 (33.23–40.50)	38.50 (34.40–40.60)
SCr μmol/L at month 3	85.55 (70.13–106.23)	87.15 (73.75–104.15)
SCr μmol/L at month 6	79.00 (65.15–104.25)	79.65 (68.78–101.58)
TA-SCr (μmol/L)	84.06 (65.33–107.54)	87.94 (70.05–104.79)
Peak SCr (μmol/L)	95.00 (74.03–130.03)	100.00 (80.30–120.80)
eGFR (ml/min/1.73 m2) at month 3	75.30 (56.38–98.19)	72.17 (55.99–96.98)
eGFR (ml/min/1.73 m2) at month 6	81.78 (59.73–106.02)	80.90 (64.64–107.02)
TA-eGFR (ml/min/1.73 m2)	78.91 (57.43–105.00)	76.87 (57.37–105.21)
Minimum eGFR (ml/min/1.73 m2)	67.66 (45.96–88.63)	67.49 (47.27–86.18
UPE (g/d) at month 3	0.81 (0.37–1.63)	0.84 (0.55–1.52)
UPE (g/d) at month 6	0.63 (0.27–1.38)	0.63 (0.32–1.55)
TA-UPE (g/d)	0.80 (0.51–1.72)	0.82 (0.53–1.84)
Peak UPE (g/d)	1.90 (1.13–3.41)	1.82 (1.11–3.39)

Values are presented as the means ± standard deviation (SD) if the variables showed a normal distribution and as medians (IQR) if the variables did not show a normal distribution; n (%) was used for the categorical variables.

SBP: systolic blood pressure; DBP: diastolic blood pressure; MAP: mean arterial blood pressure; SCr: serum creatinine; BUN: blood urea nitrogen; eGFR: estimated glomerular filtration rate; UA: serum uric acid; Hb: hemoglobin; ALB: serum albumin; UPE: 24-h urinary protein excretion; uRBC/HP: hematuria; TA-ALB: time-averaged serum albumin; TA-SCr: time-averaged serum creatinine; TA-eGFR: time-averaged eGFR; TA-UPE: time-averaged 24-h urinary protein excretion; Peak UPE: maximum level of UPE during follow-up; Peak SCr: maximum level of SCr during follow-up; Minimum ALB: minimum level of ALB; Minimum eGFR: minimum eGFR during follow-up; UPE ratio: (UPE at follow-up—baseline UPE) / baseline UPE.

### Treatment and Patient Response in the Primary Cohort

Most of the patients were treated according to the accepted standards at our center as follows: (1) full doses of angiotensin-converting enzyme inhibitors (ACEis) and/or angiotensin receptor blockers (ARBs) were recommended for all the patients with proteinuria or hypertension; (2) steroids were used in cases of massive proteinuria (> 1 g/d); and (3) other immunosuppressive agents were considered in patients with impaired kidney function or rapidly progressing kidney function decline (an increase in SCr > 15% in the year prior to entry into the trial) [19].

CR and PR were achieved in 191 (29.1%) and 298 (45.3%) patients, respectively. However, 168 patients (25.6%) exhibited a minimal response or NR. Finally, a total of 489 patients with IgAN achieved remission within 6 months of the start of therapy. Additionally, 15.5% of the remission patients experienced relapse during the follow-up. During the median follow-up period of 66 months, 6 patients (1.4%) in the non-relapse group reached the endpoint, compared with 22 (29.6%) in the relapse group. Ultimately, 9.5% of the 657 included patients reached the endpoint during the follow-up period in this study. There was no statistical difference microscopic hematuria among complete remission patients, partial remission patients and non-response patients by ANOVA-test. More non-remission patients had received ACEIs/ARBs than remission patients (including complete remission and partial remission patients) having received. And the study revealed that patients having been treated with ACEIs/ARBs would relapse more promptly.

### Kidney Progression and Risk Factors in the Primary Cohort

Clinical variables at the onset of disease and during follow-up were tested for association with renal survival. In the univariate Cox regression analysis, 11 variables were found to be risk factors of composite kidney failure events, namely, non-remission, relapse, ALB at month 6, TA-ALB, SCr at month 3, SCr at month 6, TA-SCr, eGFR at month 3, eGFR at month 6, TA-eGFR, and UPE at month 3. All variables that passed the univariate test were used in the multivariate analysis. Two multivariate Cox regression models were created, as shown in Tables 2 and 3. In Table 2, age, TA-ALB, and TA-SCr were independently predictive of IgA nephropathy progression. PI of Table 2 = 0.041*age (years)-0.124*TA-ALB(g/L)+0.015 *TA-SCr (μmol/L). After adjusting for the effects of age, gender, SCr, eGFR, ALB, and UPE, individuals who experienced relapse had a significantly increased risk of renal progression [hazard ratio (HR) = 28.268; 95% confidence interval (CI): 1.962–407.223; P = 0.014], which was shown in Table 3. In the adjusted model, individuals who experienced relapse had a 27.268-fold greater risk of renal progression compared with non-relapse individuals. PI of Table 3 = 3.342*(1 if non-relapse; 2 if relapse)-0.268*TA-ALB(g/L)+0.019 *TA-SCr (μmol/L).

**Table 2:** Factors that were found to affect long-term prognosis in IgAN patients in the multivariate Cox regression analysis of the Primary Cohort (n = 657).

Characteristics	Univariate analysis			Multivariate analysis			
	HR	95% CI	P value	Coeffecient	HR	95% CI	P value
Age (years)			0.063	0.041	1.042		0.03
Gender: male (vs. female)			0.943				0.67
TA-ALB (g/L)	0.886	0.796–0.986	0.027	-0.124	0.883	0.788–0.991	0.03
TA-eGFR (ml/min/1.73 m^2^)	0.98	0.964–0.996	0.017				0.32
TA-SCr (μmol/L)	1.011	1.004–1.017	0.001	0.015	1.015	1.006–1.024	0.001
UPE at month 3 >1 g/d (vs. <1 g/d)	2.632	1.072–6.461	0.035				0.09

NS: no significant difference

**Table 3:** Factors that were found to affect long-term prognosis in IgAN patients in the multivariate Cox regression analysis of the patients having achieved remission. (n = 489).

Characteristics	Univariate analysis				Multivariate analysis		
	HR	95% CI	P value	Coeffecient	HR	95% CI	P value
Age (years)			0.063				0.51
Gender: male (vs. female)			0.943				0.21
Relapse (vs. non-relapse)	10.51	2.173–50.835	0.003	3.342	28.268	1.962–407.223	0.01
TA-ALB (g/L)	0.886	0.796–0.986	0.027	-0.268	0.765	0.593–0.988	0.04
TA-eGFR (ml/min/1.73 m^2^)	0.98	0.964–0.996	0.017				0.66
TA-SCr (μmol/L)	1.011	1.004–1.017	0.001	0.019	1.019	1.006–1.032	0.005
UPE at month 3 >1 g/d (vs. <1 g/d)	2.615	1.066–6.419	0.035				0.68

NS: no significant difference

**Fig 2:**
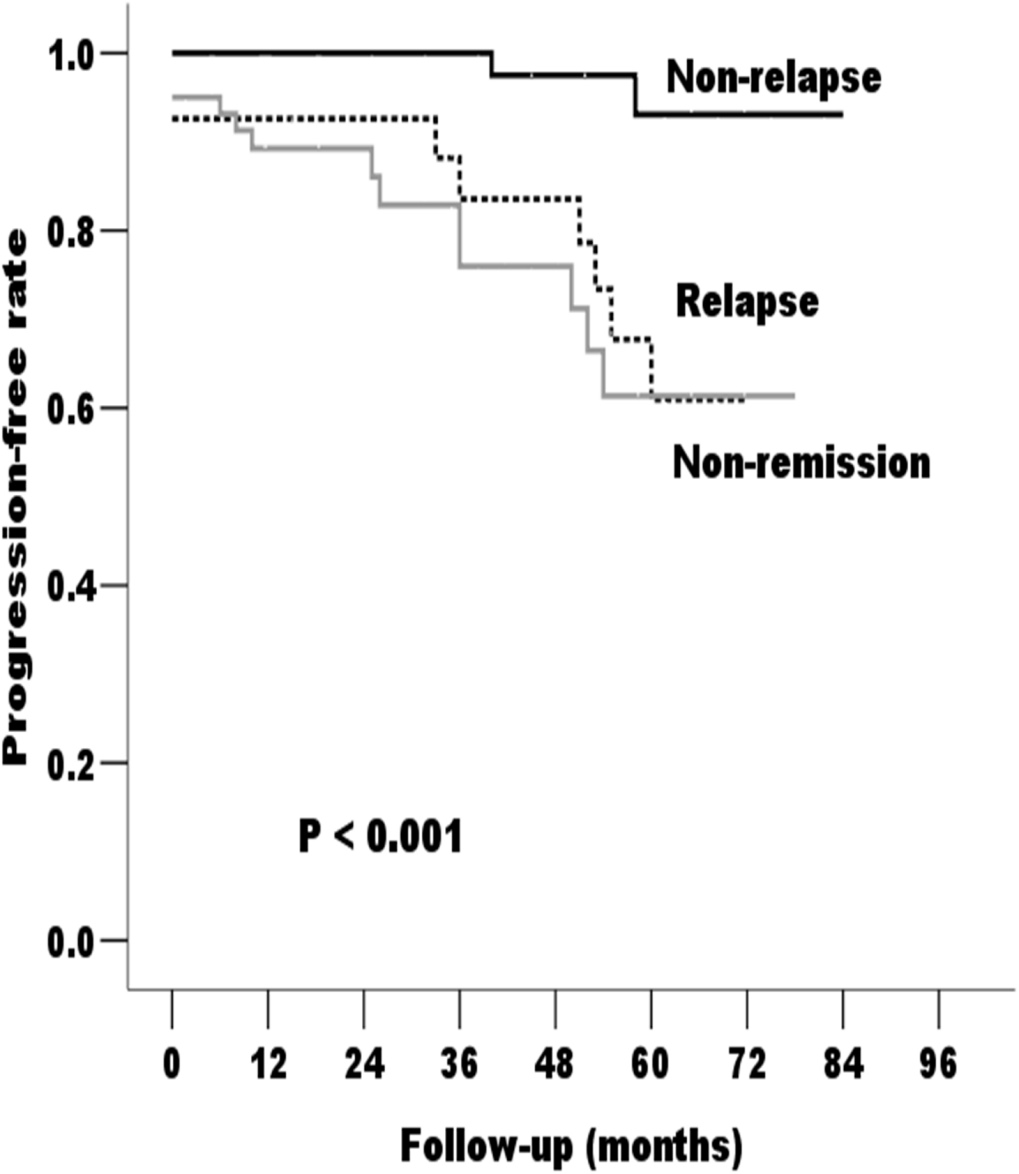
Kaplan-Meier cumulative renal progression-free survival rate according to the incidence of non-relapse, relapse, and non-remission during the follow-up period of the Primary Cohort. The non-relapse patients had significantly longer progression-free times compared with the relapse and non-remission patients (P < 0.05).

Based on the Kaplan-Meier analyses, the actual renal survival rates according to the patients’ response to treatment are plotted in Fig 2. As illustrated in Fig 2, all patients who experienced relapse after achieving remission, regardless of the starting point, had an unfavorable survival rate of non-progression.

### Predictors of renal relapse in IgAN patients who achieved remission following six months of therapy in the Primary Cohort

We had analyzed the association between the therapeutic regimens and renal relapse, but there is no significant correlation. Microscopic hematuria at baseline, the third month and the sixth month and therapeutic regimens were not associated with relapse. In this study, we had investigated pathological features such as mesangial hypercellularity, endocapillary proliferation, segmental sclerosis, tubular atrophy and interstitial fibrosis in relapse group and the non-relapse group, and there were no significant differences. Furthermore, we also evaluated fluorescence staining of renal biopsy for renal relapse, and there is no significant difference. Both univariate and multivariate Cox analyses were performed to evaluate the impact of the potential predictors of renal relapse. As shown in Table 4, in the univariate analyses, gender and baseline DBP were statistically significant. The factors that were significantly correlated with progression in the univariate analysis were further evaluated in the multivariate analysis. The results revealed that both gender and baseline DBP were independently associated with renal relapse. The adjusted multivariate Cox analysis model indicated that each 1-mmHg increase in the baseline DBP was associated with a 4.5% increase in the risk of renal relapse, and male patients had a 3.324-fold greater risk of experiencing relapse compared with female patients. We also compared duration from starting the therapy to renal relapse between female and male patients, as well as patients with DBP > 80mm Hg and DBP < 80 mm Hg. The results showed that male patients with DBP > 80mm Hg would experience relapse more promptly (Tables 5 and 6). PI of relapse = 1.201*(1 if female; 2 if male)+0.044*DBP(mmHg).

**Table 4:** Factors that were found to affect relapse of IgAN patients in the univariate and multivariate Cox regression analyses of the Primary Cohort.

Characteristics	Univariate analysis				Multivariate analysis		
	HR	95% CI	P value	Coeffecient	HR	95% CI	P value
Age (years)			0.088				0.25
Gender: male (vs. female)	3.62	1.562–8.378	0.003	1.201	3.32	1.221–9.051	0.02
ACEI/ARB (vs Steroids or Immunosuppressors)			0.697				
DBP (mmHg)	1.04	1.001–1.070	0.045	0.044	1.05	1.003–1.089	0.04
TA-SCr (μmol/L)			0.055				0.58

NS: no significant difference

**Table 5:** Comparison of duration from starting the therapy to renal relapse in patients with DBP>80 mmHg and DBP<80 mmHg of the Primary Cohort.

Time	DBP>80 mmHg	DBP<80 mmHg	P
	N = 47	N = 29	
Time from start of therapy to relapse (months)	24.18±2.13	30.70±1.83	0.029

**Table 6:** Comparison of duration from starting the therapy to renal relapse in male patients and female patients of the Primary Cohort.

Time	Male	Female	P
	N = 50	N = 26	
Time from start of therapy to relapse (months)	25.12±1.88	30.25±1.97	0.028

### Prognostic indexes

**Fig 3:**
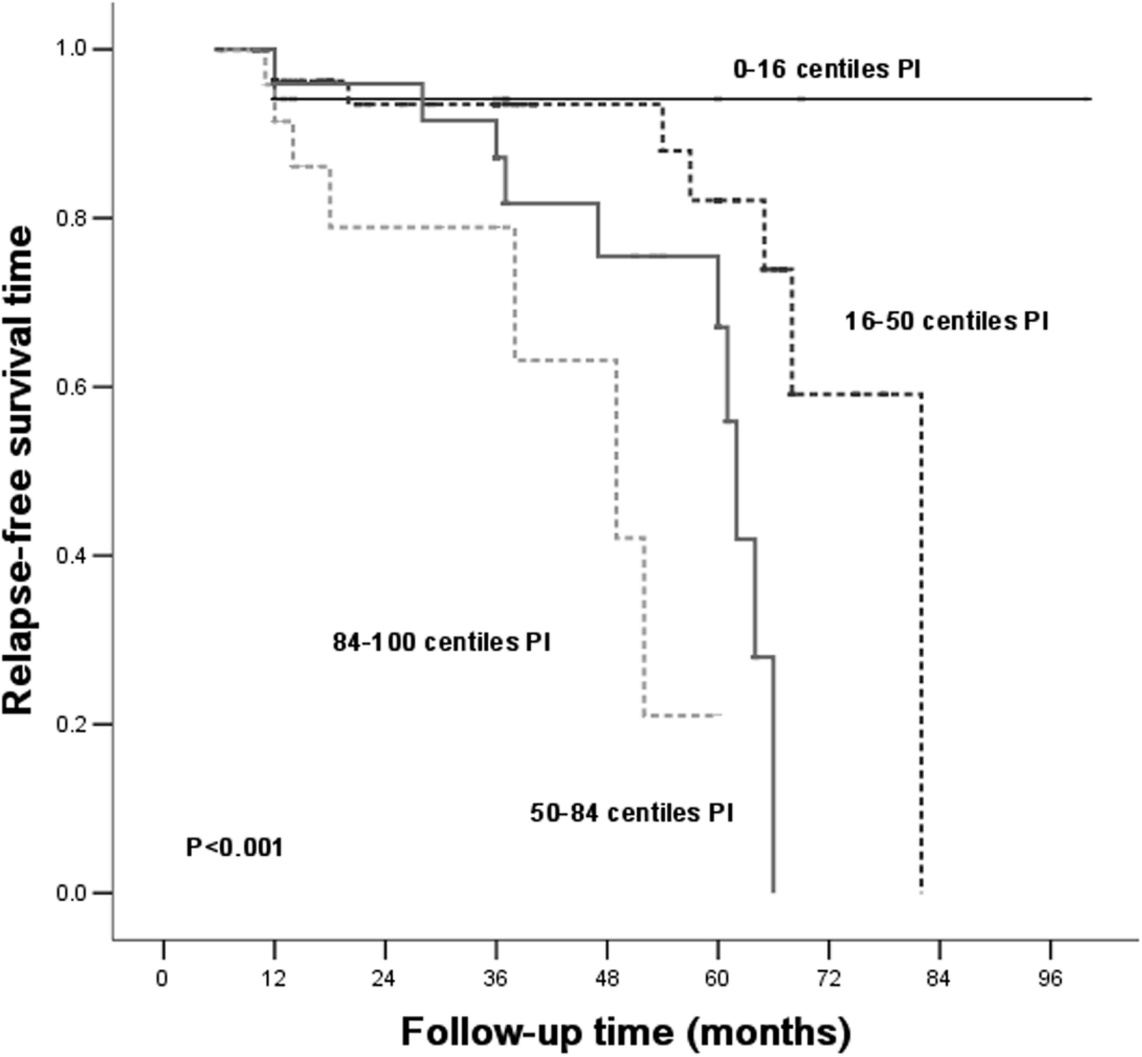
Kaplan-Meier cumulative IgAN relapse-free rate according to the PI based on Table 4, relapse-free survival analysis of the Primary Cohort.

Patients were divided into different risk stratum based on PI of relapse. The observed probability for relapse-free survival was plotted and distinguished in each risk stratum (Fig 3). Patients of the 0^th^ to the 16^th^ PI based on Table 4 tend to be protected from relapsing.

### Prognostic Nomogram for PI of relapse

**Fig 4:**
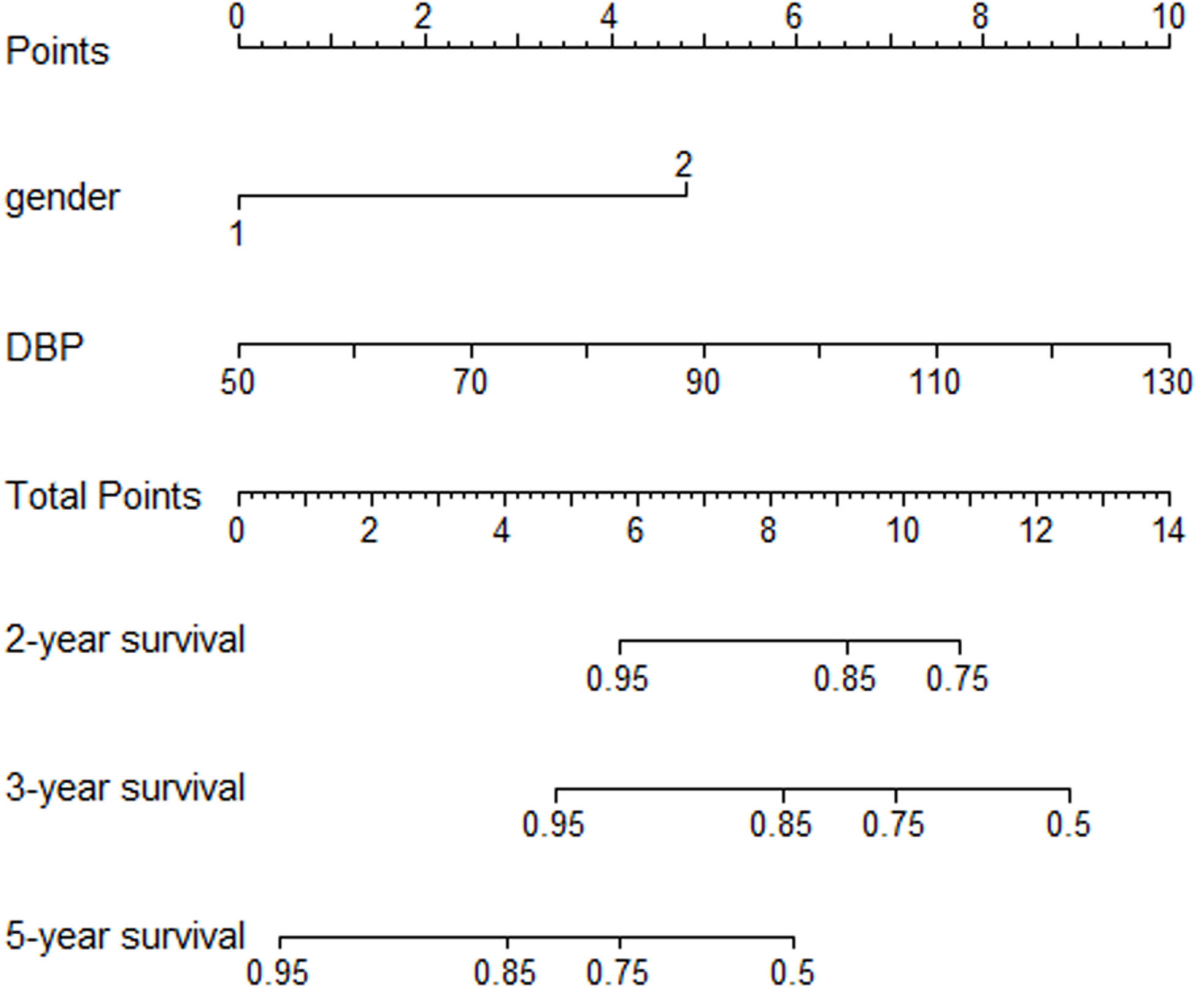
IgAN patients’ relapse nomogram. To use the nomogram, an individual patient’s value is located on each variable axis, and a line is drawn upward to determine the number of points received for each variable value. The sum of these numbers is located on the Total Points axis, and a line is drawn downward to the survival axes to determine the likelihood of 2-,3- or 5-year IgAN relapse.

The prognostic nomogram that integrated all significant independent factors for relapse in the primary cohort is shown in Fig 4. The C-index for PI of relapse prediction was 0.78 (95% CI, 0.62 to 0.93) (Table 7).

**Table 7:** Performance of the PI based on the Cox- regression for Prediction of renal outcome of the Primary Cohort.

PI	Harrell's concordance index(C-index)	95% CI of C-index
PI of Table 2	0.72	0.55–0.89
PI of Table 3	0.92	0.85–0.99
PI of relapse	0.78	0.62–0.93

**Fig 5:**
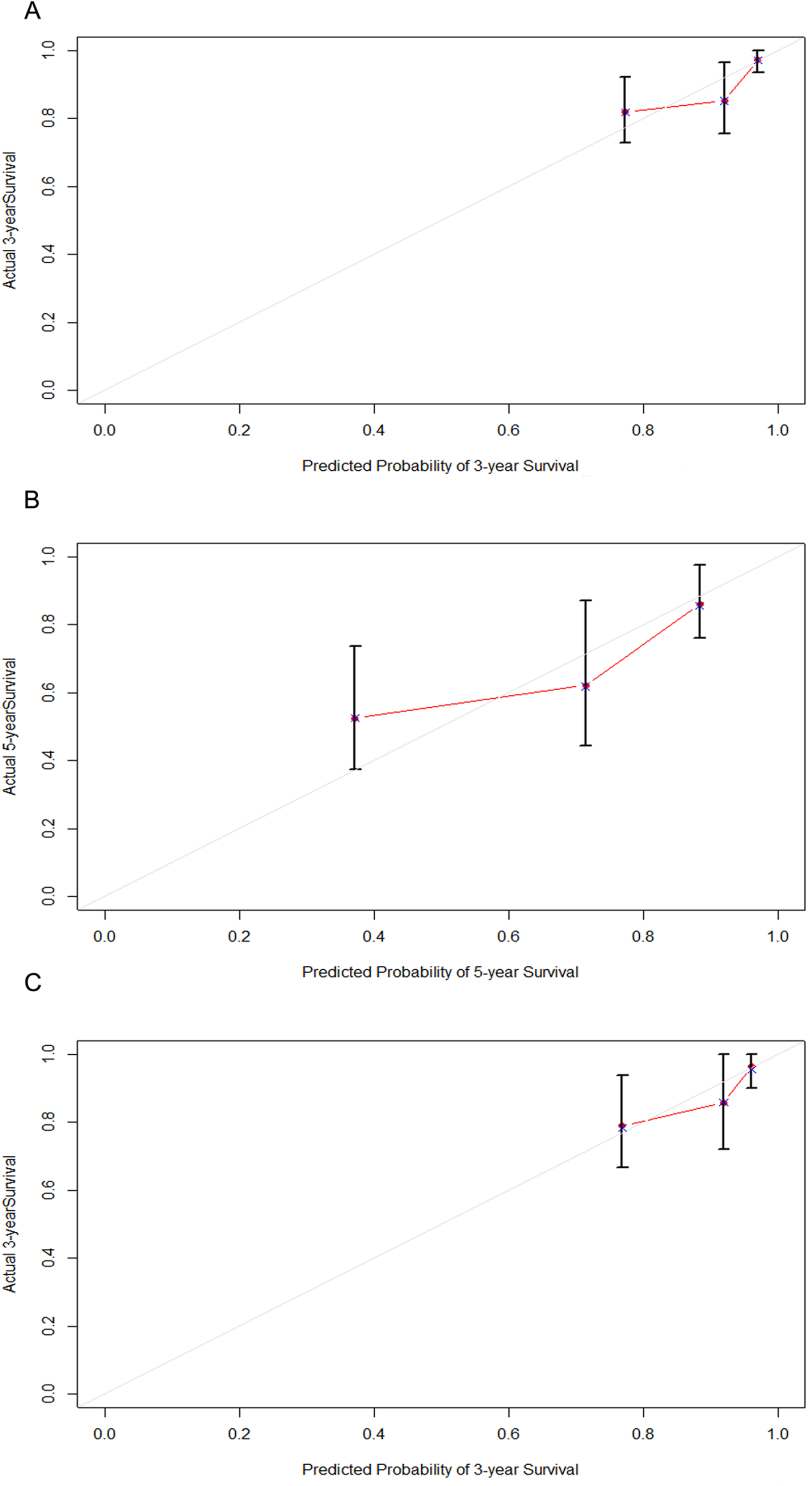
The calibration curve for predicting IgAN patients’ relapse nomogram. (A) at 3 years in the primary cohort and (B) at 5 years in the primary cohort and (C) at 3 years in the validation cohort. Nomogram-predicted probability of overall IgAN relapse is plotted on the x-axis; actual overall IgAN relapse is plotted on the y-axis.

The calibration plot for the probability of relapse-free survival at 3 or 5 year showed an optimal agreement between the prediction by nomogram and actual observation (Fig 5A and 5B).

### Validation of Predictive Accuracy of the Nomogram for PI of Relapse

In the validation cohort, the median and IQR of the follow-up time was 31 and 22–56 months, respectively. CR and PR were achieved in 52(26.1%) and 105 (52.8%) patients, respectively and 17.8% of the remission patients experienced relapse during the follow-up. The C-index of the nomogram for predicting relapse was 0.71 (95% CI, 0.68 to 0.73), and a calibration curve showed good agreement between prediction and observation in the probability of 3-year relapse-free survival (Fig 5C).

## Discussion

IgAN is a progressive disease with a high variability of clinical presentations and outcomes [2]. Studies have demonstrated that the persisting remission state of proteinuria, whether spontaneous or induced by therapy, is associated with a good outcome in IgAN patients [8, 13, 20]. However, to date, few studies have addressed remission and relapse characteristics in IgAN patients.

Although the concept of “relapse” is not commonly associated with IgAN, its clinical significance and prognostic value in other forms of GN have been demonstrated [21–26]. In a study based on a southeastern U.S. pediatric cohort with severe lupus nephritis, Keisha L et al. demonstrated that remission, whether complete or partial, is associated with improved kidney survival in children with lupus nephritis, as well as that renal relapse is a strong predictor of progression to ESRD [24]. A retrospective review of adult minimal-change disease patients indicated that at least one relapse occurred in 73% of the patients and that 28% experienced frequent relapses. Furthermore, patients who frequently relapsed were more likely to progress to ESRD [27].

In clinical practice, many IgAN patients have frequent relapses or are treatment-resistant. The kidneys sustain some chronic damage with each relapse that may culminate in CKD or, eventually, ESRD, suggesting that there may be a high incidence of renal relapse among patients who experience IgAN progression. Our previous study demonstrated that 16.5% of patients who achieved remission within 6 months of therapy experienced renal deterioration within the mean follow-up time of 49 months [28]. Other studies have confirmed that 1%-2% of IgAN patients progress to ESRD within 1 year of diagnosis [4, 29], and approximately 40% of patients will ultimately develop ESRD within 20 years [1, 4, 5]. Until now, few studies have investigated the association of different therapeutic responses, such as remission and relapse, with the risk of renal function decline. Therefore, we speculated that relapse may be a potential predictor of IgAN prognostic outcomes, as demonstrated in the present study.

In this study, we found that relapse or age, ALB, and SCr were independently associated with the composite renal outcome. To our knowledge, our study is the first to identify relapse as an independent risk factor for the progression of IgAN. After adjusting for the effects of age, gender, SCr, eGFR, ALB, and UPE, individuals who experienced relapse had a significantly increased risk of renal progression.

Baseline SCr is among the most consistently reported predictors of progression [1, 10, 29, 30], but our study indicated that SCr at months 3 and 6, rather than at baseline, was significantly related to the prognosis of IgAN. TA-SCr, representing the levels of renal function during follow-up within the first year of diagnosis, was an important independent risk factor for renal progression. There are many factors that may modify the SCr value. These factors include changes in intravascular volume, comorbid conditions, and many drugs. Patients being diagnosed would be given health recommendations. Accordingly, they would make some lifestyle modification. As a result, measurement of SCr at months 3 and 6 could exclude potential bias to some extend and might be more precise for analysis.

Recently, studies have quantified the impact of proteinuria reduction in IgAN and the clinical relevance of defining CR or PR in this disease as a valuable prognostic indicator [3, 14]. Studies have also demonstrated strong associations between non-remission and accelerated renal disease progression, and proteinuria >1 g/d at month 3 was adversely related to long-term renal prognosis, similar to findings observed across multiple cohorts [4, 14, 20].

This study identified ALB during follow-up as an independent risk factor for renal outcomes in IgAN patients, quantifying the association of the decline in eGFR with ALB at month 6 and TA-ALB. This result supports our earlier finding that the TA-ALB value is a potential predictor of renal progression in patients with IgAN and that patients with a higher level of TA-ALB have excellent outcomes without progression [28]. Liu ZH et al. [13] found that baseline hypoproteinemia was an independent risk factor for an unfavorable IgAN outcome. This study revealed that to keep a relative high level of ALB was recommended in IgAN patients. Study of Jingyuan Xie and Nan Chen[9] confirmed serum ALB as a risk factor for IgAN progression. Another study based on Japanese patients also identified serum ALB as a significant risk factor for ESRD, even 1854 of the 2083 included patients had serum ALB more than 37 g/L[31].

As in previous studies[30, 32, 33], our multivariate analysis also suggested that older age at diagnosis is a strong predictor of renal progression. Relapses are important to recognize and treat. Identifying an association between related risk factors and renal relapse in IgAN is particularly helpful in understanding the progression and risk factors of IgAN based on current therapy techniques. In this study, renal relapse was significantly related to the male gender and baseline DBP. In a study based on IgAN patients with nephrotic syndrome, Jwa-Kyung Kim et al. found that female patients tended to have a more favorable probability of achieving spontaneous remission [3]. In this study, the adjusted multivariate Cox analysis model indicated that male patients had a 3.324-fold greater risk of experiencing relapse compared with female patients. The above-mentioned study implied that female patients exhibited a good response to therapy. Furthermore, many studies have demonstrated the important role of arterial blood pressure in determining IgAN prognosis [9, 11, 34]. In this study, we found that a higher baseline DBP was associated with a higher risk of experiencing renal relapse.

This is the first study to use a cohort of IgAN patients from multiple centers to assess renal outcomes among groups that exhibit different responses to therapies, including a large portion of individuals experiencing relapse. Our study may help explain the progression and risk factors of IgAN associated with current therapy techniques. This study is unique in that 903 IgAN patients who received a uniform therapy strategy were followed for up to 9 or more years. Using this robust database, we examined the impact that relapse patterns have on progression to ESRD, and we identified predictors of IgAN relapse. Several shortcomings of this study should be discussed. Most of the patients recruited came from the southern regions of China. There is also a concern regarding the strict nature of the definitions used for CR, PR, and relapse in this study. Therefore, the data for each of the three criteria requires further study in the future. Furthermore, we didn’t found significant difference between progression patients and non-progression patients in relapse group, which might partially because the study group of relapse was statistically small (76 patients experienced a relapse).

In summary, our data clearly showed that relapse was a potential predictor of prognostic outcomes in patients based on the current therapeutic regimens for IgAN. Additionally, nephritis relapse was significantly related to the male gender and baseline DBP.

## Abbreviations and Acronyms

ACEIs: angiotensin-converting enzyme inhibitors
ALB: albumin
ARBs: angiotensin receptor blockers
BUN: blood urea nitrogen
CI: confidence interval
CKD: Chronic kidney disease
C-index: concordance index
CR: complete remission
baseline diastolic blood pressure (DBP): diastolic blood pressure
eGFR: the estimated glomerular filtration rate
ESRD: end-stage renal disease
GN: glomerulonephritis
HR: hazard ratio
IgAN: IgA nephropathy
IQR: interquartile range
NR: No response
PI: prognostic index
PR: partial remission
SBP: systolic blood pressure
SCr: serum creatinine, time-averaged serum albumin (TA-ALB), time-averaged ALB
TA-eGFR: time-averaged eGFR
time-averaged serum creatinine (TA-SCr): time-averaged SCr
TA-UPE: time-averaged UPE
UPE: 24-hour urinary protein excretion
